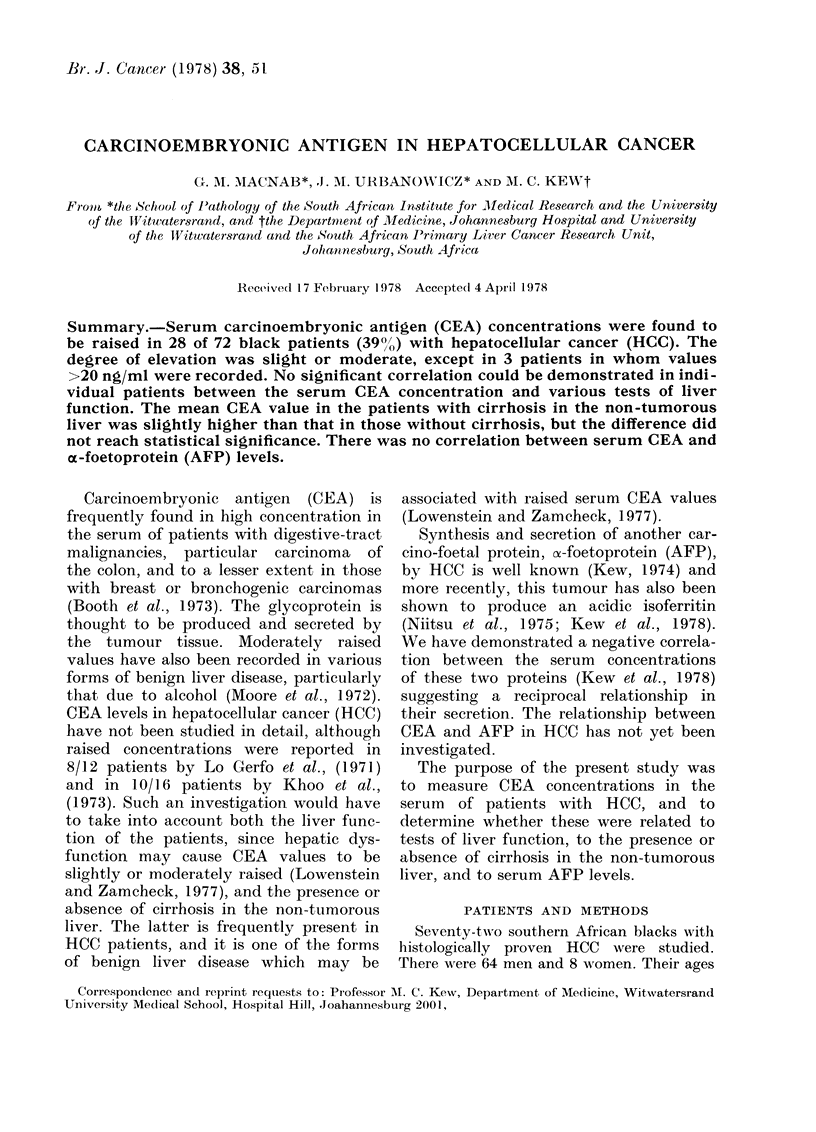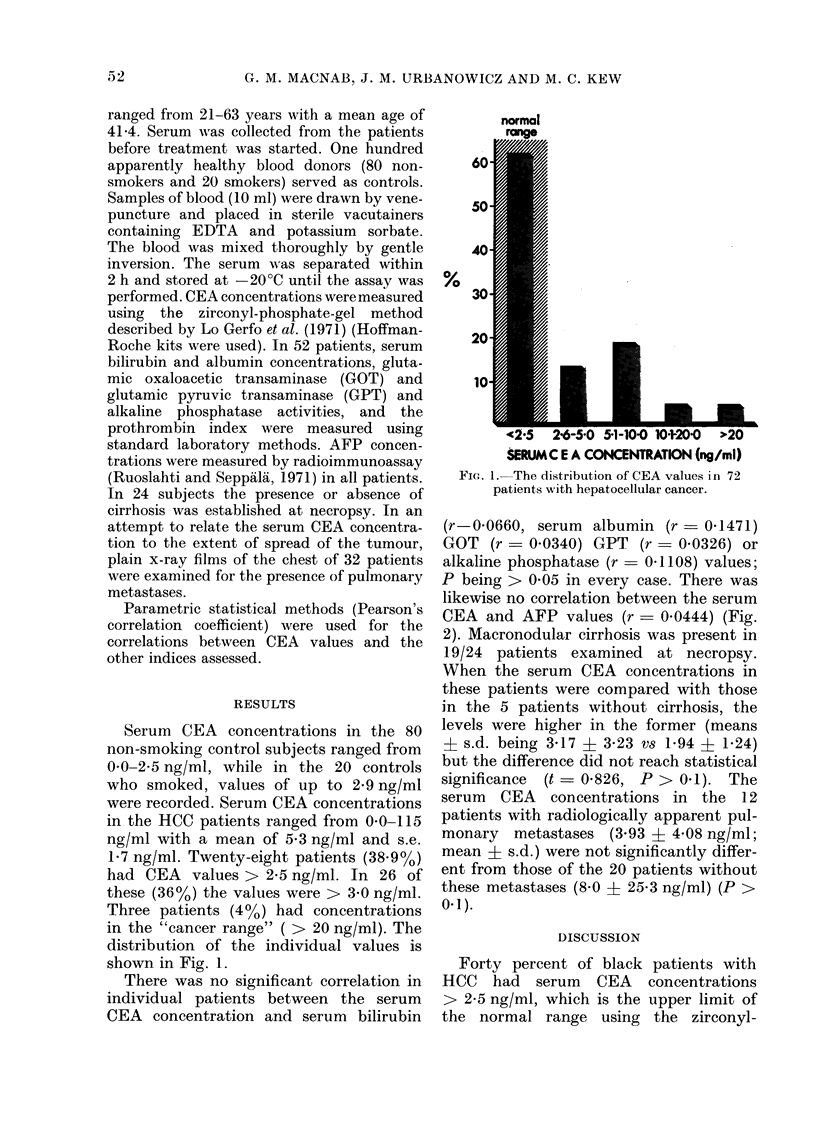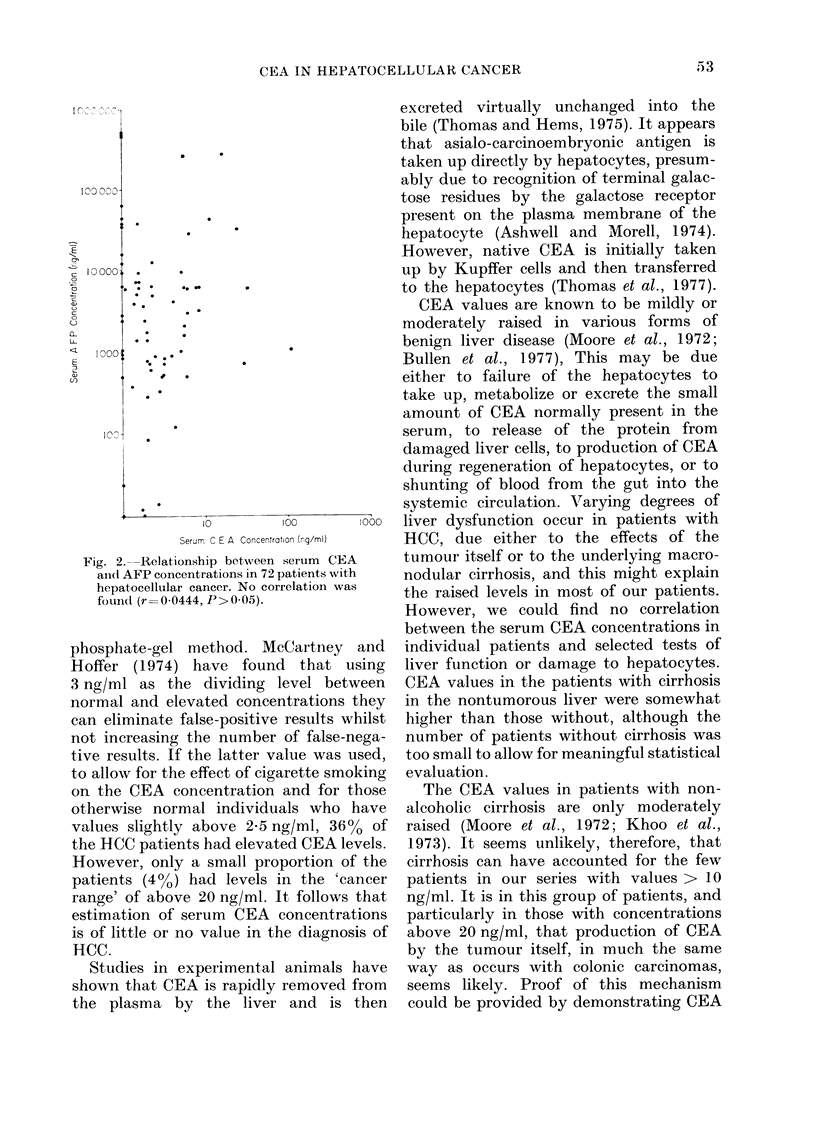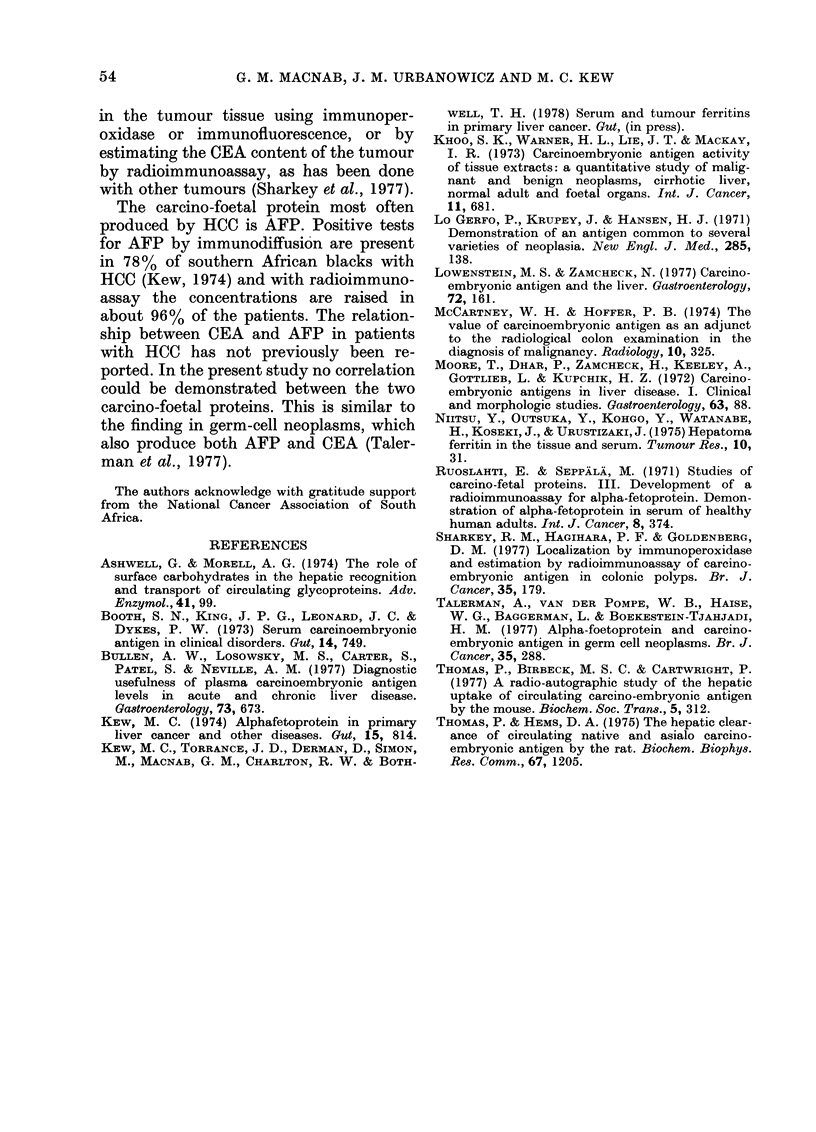# Carcinoembryonic antigen in hepatocellular cancer.

**DOI:** 10.1038/bjc.1978.162

**Published:** 1978-07

**Authors:** G. M. Macnab, J. M. Urbanowicz, M. C. Kew

## Abstract

Serum carcinoembryonic antigen (CEA) concentrations were found to be raised in 28 of 72 black patients (39%) with hepatocellular cancer (HCC). The degree of elevation was slight or moderate, except in 3 patients in whom values greater than 20 ng/ml were recorded. No significant correlation could be demonstrated in individual patients between the serum CEA concentration and various tests of liver function. The mean CEA value in the patients with cirrhosis in the non-tumorous liver was slightly higher than that in those without cirrhosis, but the difference did not reach statistical significance. There was no correlation between serum CEA and alpha-foetoprotein (AFP) levels.


*Br. J. Cancer* (1978) **38**, 51

### CARCINOEMBRYONIC ANTIGEN IN HEPATOCELLULAR CANCER

G. M. MACNAB*, J. M. URBANOWICZ* AND M. C. KEW†

*From *the School of Pathology of the South African Institute for Medical Research and the University of the Witwatersrand, and †the Department of Medicine, Johannesburg Hospital and University of the Witwatersrand and the South African Primary Liver Cancer Research Unit, Johannesburg, South Africa*

Received 17 February 1978 Accepted 4 April 1978

**Summary.—Serum carcinoembryonic antigen (CEA) concentrations were found to be raised in 28 of 72 black patients (39%) with hepatocellular cancer (HCC). The degree of elevation was slight or moderate, except in 3 patients in whom values >20 ng/ml were recorded. No significant correlation could be demonstrated in individual patients between the serum CEA concentration and various tests of liver function. The mean CEA value in the patients with cirrhosis in the non-tumorous liver was slightly higher than that in those without cirrhosis, but the difference did not reach statistical significance. There was no correlation between serum CEA and α-foetoprotein (AFP) levels.**

Carcinoembryonic antigen (CEA) is frequently found in high concentration in the serum of patients with digestive-tract malignancies, particular carcinoma of the colon, and to a lesser extent in those with breast or bronchogenic carcinomas (Booth *et al.*, 1973). The glycoprotein is thought to be produced and secreted by the tumour tissue. Moderately raised values have also been recorded in various forms of benign liver disease, particularly that due to alcohol (Moore *et al.*, 1972). CEA levels in hepatocellular cancer (HCC) have not been studied in detail, although raised concentrations were reported in 8/12 patients by Lo Gerfo *et al.*, (1971) and in 10/16 patients by Khoo *et al.*, (1973). Such an investigation would have to take into account both the liver function of the patients, since hepatic dysfunction may cause CEA values to be slightly or moderately raised (Lowenstein and Zamcheck, 1977), and the presence or absence of cirrhosis in the non-tumorous liver. The latter is frequently present in HCC patients, and it is one of the forms of benign liver disease which may be associated with raised serum CEA values (Lowenstein and Zamcheck, 1977).

Synthesis and secretion of another carcino-foetal protein, α-foetoprotein (AFP), by HCC is well known (Kew, 1974) and more recently, this tumour has also been shown to produce an acidic isoferritin (Niitsu *et al.*, 1975; Kew *et al.*, 1978). We have demonstrated a negative correlation between the serum concentrations of these two proteins (Kew *et al.*, 1978) suggesting a reciprocal relationship in their secretion. The relationship between CEA and AFP in HCC has not yet been investigated.

The purpose of the present study was to measure CEA concentrations in the serum of patients with HCC, and to determine whether these were related to tests of liver function, to the presence or absence of cirrhosis in the non-tumorous liver, and to serum AFP levels.

### PATIENTS AND METHODS

Seventy-two southern African blacks with histologically proven HCC were studied. There were 64 men and 8 women. Their ages

Correspondence and reprint requests to: Professor M. C. Kew, Department of Medicine, Witwatersrand University Medical School, Hospital Hill, Joahannesburg 2001, ranged from 21–63 years with a mean age of 41·4. Serum was collected from the patients before treatment was started. One hundred apparently healthy blood donors (80 non-smokers and 20 smokers) served as controls. Samples of blood (10 ml) were drawn by venepuncture and placed in sterile vacutainers containing EDTA and potassium sorbate. The blood was mixed thoroughly by gentle inversion. The serum was separated within 2 h and stored at −20°C until the assay was performed. CEA concentrations were measured using the zirconyl-phosphate-gel method described by Lo Gerfo *et al.* (1971) (Hoffman-Roche kits were used). In 52 patients, serum bilirubin and albumin concentrations, glutamic oxaloacetic transaminase (GOT) and glutamic pyruvic transaminase (GPT) and alkaline phosphatase activities, and the prothrombin index were measured using standard laboratory methods. AFP concentrations were measured by radioimmunoassay (Ruoslahti and Seppälä, 1971) in all patients. In 24 subjects the presence or absence of cirrhosis was established at necropsy. In an attempt to relate the serum CEA concentration to the extent of spread of the tumour, plain x-ray films of the chest of 32 patients were examined for the presence of pulmonary metastases.

Parametric statistical methods (Pearson's correlation coefficient) were used for the correlations between CEA values and the other indices assessed.

### RESULTS

Serum CEA concentrations in the 80 non-smoking control subjects ranged from 0·0–2·5 ng/ml, while in the 20 controls who smoked, values of up to 2·9 ng/ml were recorded. Serum CEA concentrations in the HCC patients ranged from 0·0–115 ng/ml with a mean of 5·3 ng/ml and s.e. 1·7 ng/ml. Twenty-eight patients (38·9%) had CEA values $>$ 2·5 ng/ml. In 26 of these (36%) the values were $>$ 3·0 ng/ml. Three patients (4%) had concentrations in the "cancer range" ( $>$ 20 ng/ml). The distribution of the individual values is shown in Fig. 1.

FIG. 1.—The distribution of CEA values in 72 patients with hepatocellular cancer.

There was no significant correlation in individual patients between the serum CEA concentration and serum bilirubin ($r$—0·0660, serum albumin ($r$ = 0·1471) GOT ($r$ = 0·0340) GPT ($r$ = 0·0326) or alkaline phosphatase ($r$ = 0·1108) values; $P$ being $>$ 0·05 in every case. There was likewise no correlation between the serum CEA and AFP values ($r$ = 0·0444) (Fig. 2). Macronodular cirrhosis was present in 19/24 patients examined at necropsy. When the serum CEA concentrations in these patients were compared with those in the 5 patients without cirrhosis, the levels were higher in the former (means ± s.d. being 3·17 ± 3·23 *vs* 1·94 ± 1·24) but the difference did not reach statistical significance ($t$ = 0·826, $P > 0{\cdot}1$). The serum CEA concentrations in the 12 patients with radiologically apparent pulmonary metastases (3·93 ± 4·08 ng/ml; mean ± s.d.) were not significantly different from those of the 20 patients without these metastases (8·0 ± 25·3 ng/ml) ($P >$ 0·1).

### DISCUSSION

Forty percent of black patients with HCC had serum CEA concentrations $>$ 2·5 ng/ml, which is the upper limit of the normal range using the zirconyl-

Fig. 2.—Relationship between serum CEA and AFP concentrations in 72 patients with hepatocellular cancer. No correlation was found ($r = 0{\cdot}0444$, $P > 0{\cdot}05$).

phosphate-gel method. McCartney and Hoffer (1974) have found that using 3 ng/ml as the dividing level between normal and elevated concentrations they can eliminate false-positive results whilst not increasing the number of false-negative results. If the latter value was used, to allow for the effect of cigarette smoking on the CEA concentration and for those otherwise normal individuals who have values slightly above 2·5 ng/ml, 36% of the HCC patients had elevated CEA levels. However, only a small proportion of the patients (4%) had levels in the 'cancer range' of above 20 ng/ml. It follows that estimation of serum CEA concentrations is of little or no value in the diagnosis of HCC.

Studies in experimental animals have shown that CEA is rapidly removed from the plasma by the liver and is then excreted virtually unchanged into the bile (Thomas and Hems, 1975). It appears that asialo-carcinoembryonic antigen is taken up directly by hepatocytes, presumably due to recognition of terminal galactose residues by the galactose receptor present on the plasma membrane of the hepatocyte (Ashwell and Morell, 1974). However, native CEA is initially taken up by Kupffer cells and then transferred to the hepatocytes (Thomas *et al.*, 1977).

CEA values are known to be mildly or moderately raised in various forms of benign liver disease (Moore *et al.*, 1972; Bullen *et al.*, 1977), This may be due either to failure of the hepatocytes to take up, metabolize or excrete the small amount of CEA normally present in the serum, to release of the protein from damaged liver cells, to production of CEA during regeneration of hepatocytes, or to shunting of blood from the gut into the systemic circulation. Varying degrees of liver dysfunction occur in patients with HCC, due either to the effects of the tumour itself or to the underlying macronodular cirrhosis, and this might explain the raised levels in most of our patients. However, we could find no correlation between the serum CEA concentrations in individual patients and selected tests of liver function or damage to hepatocytes. CEA values in the patients with cirrhosis in the nontumorous liver were somewhat higher than those without, although the number of patients without cirrhosis was too small to allow for meaningful statistical evaluation.

The CEA values in patients with non-alcoholic cirrhosis are only moderately raised (Moore *et al.*, 1972; Khoo *et al.*, 1973). It seems unlikely, therefore, that cirrhosis can have accounted for the few patients in our series with values $> 10$ ng/ml. It is in this group of patients, and particularly in those with concentrations above 20 ng/ml, that production of CEA by the tumour itself, in much the same way as occurs with colonic carcinomas, seems likely. Proof of this mechanism could be provided by demonstrating CEA

in the tumour tissue using immunoperoxidase or immunofluorescence, or by estimating the CEA content of the tumour by radioimmunoassay, as has been done with other tumours (Sharkey *et al.*, 1977).

The carcino-foetal protein most often produced by HCC is AFP. Positive tests for AFP by immunodiffusion are present in 78% of southern African blacks with HCC (Kew, 1974) and with radioimmunoassay the concentrations are raised in about 96% of the patients. The relationship between CEA and AFP in patients with HCC has not previously been reported. In the present study no correlation could be demonstrated between the two carcino-foetal proteins. This is similar to the finding in germ-cell neoplasms, which also produce both AFP and CEA (Talerman *et al.*, 1977).

The authors acknowledge with gratitude support from the National Cancer Association of South Africa.

### REFERENCES

ASHWELL, G. & MORELL, A. G. (1974) The role of surface carbohydrates in the hepatic recognition and transport of circulating glycoproteins. *Adv. Enzymol.*, **41**, 99.

BOOTH, S. N., KING, J. P. G., LEONARD, J. C. & DYKES, P. W. (1973) Serum carcinoembryonic antigen in clinical disorders. *Gut*, **14**, 749.

BULLEN, A. W., LOSOWSKY, M. S., CARTER, S., PATEL, S. & NEVILLE, A. M. (1977) Diagnostic usefulness of plasma carcinoembryonic antigen levels in acute and chronic liver disease. *Gastroenterology*, **73**, 673.

KEW, M. C. (1974) Alphafetoprotein in primary liver cancer and other diseases. *Gut*, **15**, 814.

KEW, M. C., TORRANCE, J. D., DERMAN, D., SIMON, M., MACNAB, G. M., CHARLTON, R. W. & BOTHWELL, T. H. (1978) Serum and tumour ferritins in primary liver cancer. *Gut*, (in press).

KHOO, S. K., WARNER, H. L., LIE, J. T. & MACKAY, I. R. (1973) Carcinoembryonic antigen activity of tissue extracts: a quantitative study of malignant and benign neoplasms, cirrhotic liver, normal adult and foetal organs. *Int. J. Cancer*, **11**, 681.

LO GERFO, P., KRUPEY, J. & HANSEN, H. J. (1971) Demonstration of an antigen common to several varieties of neoplasia. *New Engl. J. Med.*, **285**, 138.

LOWENSTEIN, M. S. & ZAMCHECK, N. (1977) Carcinoembryonic antigen and the liver. *Gastroenterology*, **72**, 161.

MCCARTNEY, W. H. & HOFFER, P. B. (1974) The value of carcinoembryonic antigen as an adjunct to the radiological colon examination in the diagnosis of malignancy. *Radiology*, **10**, 325.

MOORE, T., DHAR, P., ZAMCHECK, H., KEELEY, A., GOTTLIEB, L. & KUPCHIK, H. Z. (1972) Carcinoembryonic antigens in liver disease. I. Clinical and morphologic studies. *Gastroenterology*, **63**, 88.

NIITSU, Y., OUTSUKA, Y., KOHGO, Y., WATANABE, H., KOSEKI, J., & URUSTIZAKI, J. (1975) Hepatoma ferritin in the tissue and serum. *Tumour Res.*, **10**, 31.

RUOSLAHTI, E. & SEPPÄLÄ, M. (1971) Studies of carcino-fetal proteins. III. Development of a radioimmunoassay for alpha-fetoprotein. Demonstration of alpha-fetoprotein in serum of healthy human adults. *Int. J. Cancer*, **8**, 374.

SHARKEY, R. M., HAGIHARA, P. F. & GOLDENBERG, D. M. (1977) Localization by immunoperoxidase and estimation by radioimmunoassay of carcinoembryonic antigen in colonic polyps. *Br. J. Cancer*, **35**, 179.

TALERMAN, A., VAN DER POMPE, W. B., HAISE, W. G., BAGGERMAN, L. & BOEKESTEIN-TJAHJADI, H. M. (1977) Alpha-foetoprotein and carcinoembryonic antigen in germ cell neoplasms. *Br. J. Cancer*, **35**, 288.

THOMAS, P., BIRBECK, M. S. C. & CARTWRIGHT, P. (1977) A radio-autographic study of the hepatic uptake of circulating carcino-embryonic antigen by the mouse. *Biochem. Soc. Trans.*, **5**, 312.

THOMAS, P. & HEMS, D. A. (1975) The hepatic clearance of circulating native and asialo carcinoembryonic antigen by the rat. *Biochem. Biophys. Res. Comm.*, **67**, 1205.